# Doses of chloroquine in the treatment of malaria by *Plasmodium vivax* in patients between 2 and 14 years of age from the Brazilian Amazon basin

**DOI:** 10.1186/s12936-019-3072-8

**Published:** 2019-12-21

**Authors:** Luann Wendel Pereira de Sena, Amanda Gabryelle Nunes Cardoso Mello, Michelle Valéria Dias Ferreira, Marcieni Andrade de Ataide, Rosa Maria Dias, José Luiz Fernandes Vieira

**Affiliations:** Pharmacy Faculty, Para Federal University, Campus Universitario do Guama, Augusto Correa Street 01, Belem, Para 66074740 Brazil

**Keywords:** Malaria, Chloroquine, Pharmacokinetics

## Abstract

**Background:**

A total dose of chloroquine of 25 mg/kg is recommended by the World Health Organization (WHO) to treat malaria by *Plasmodium vivax.* In several endemic areas, including the Brazilian Amazon basin, anti-malarial drugs are dispensed in small plastic bags at a dosing regimen based on age. This practice can lead to suboptimal dosing of the drug, which can impact treatment outcomes. The aim of the present study was to estimate the extent of sub-dosing of chloroquine in children and adolescents with vivax malaria using an age-based dose regimen, in addition to investigating the influence of age on the plasma concentrations of chloroquine and desethylchloroquine.

**Methods:**

A study of cases was conducted with male patients with a confirmed infection by *P. vivax*, ages 2 to 14 years, using a combined regimen of chloroquine and primaquine. Height, weight and body surface area were determined at admission on the study. The total dose of chloroquine administered was estimated based on the weight and on the body surface area of the study patients. Chloroquine and desethylchloroquine were measured on Day 7 in each patient included in the study by a high-performance liquid chromatographic method with fluorescence detection.

**Results:**

A total of 81 patients were enrolled and completed the study. The median age was 9 years (2–14 years). All patients presented negative blood smears at 42 days follow-up. The total dose of chloroquine ranged from 13.1 to 38.1 mg/kg. The percentage of patients with a total dose of the drug below 25 mg/kg ranged from 29.4 to 63.6%. The total dose of chloroquine administered based on BSA ranged from 387 to 1079 mg/m^2^, increasing with age. Plasma chloroquine concentrations ranged from 107 to 420 ng/ml, increasing with age. For desethylchloroquine, the plasma concentrations ranged from 167 to 390 ng/ml, with similar values among age-groups.

**Conclusion:**

The data demonstrated the widespread exposure of children and adolescents to suboptimal doses of chloroquine in the endemic area investigated.

## Background

Malaria caused by *Plasmodium vivax* remains a relevant public health issue in the Brazilian Amazon Basin, with approximately 130,000 cases reported annually [1]. The standard treatment consists of the concurrent administration of chloroquine and primaquine. Chloroquine is a 4-aminoquinoline effective against the asexual blood stages of *P. vivax*; the usual dose to treat the infection is approximately 25 mg/kg administered in a 3-day course [2]. The oral bioavailability of chloroquine tablets is estimated to be 89% in healthy volunteers. The drug presents a large volume of distribution, a long elimination half-life of 20–60 days and is dealkylated by 30–50% in the liver to produce desethylchloroquine and other metabolites [3, 4].

There are consistent reports of decreasing chloroquine efficacy in the referred endemic area [5, 6]. In children and adolescents, a probable cause is suboptimal treatment with the drug, which can delay the clearance of parasites and result in recurrent parasitaemia thus contributing to resistance. Moreover, sub dosing can increase the risk of developing severe signs and symptoms of vivax malaria estimated at 4.7/10.000 of cases in this endemic setting [2, 7–11]. Therefore, the doses of anti-malarial drugs should be carefully estimated in children and adolescents.

The dosing regimen of drugs for children is based on age, weight (mg/kg) or body surface area (BSA) [12, 13]. The latter is one of the most significant indexes of physiological functions in paediatric practice and child health, it is based on the assumption that metabolic processes in humans are constant when expressed as a function of BSA [14, 15]. The index is often preferable to calculate the dosing regimen in children over 2 years of age and has been successful for decades in the use of chemotherapy to treat cancer [14–16, 21]. BSA-based dosage is not a routine practice to estimate chloroquine doses in children with *P. vivax*, because there are limitations in endemic areas for accurate determination of both height and weight [2]. In fact, the World Health Organization (WHO) recommends the administration of anti-malarial drugs as a standard dose per mg/kg for all patients, including young children and adolescents. In areas where *P. vivax* is sensitive, a total dose of chloroquine of 25 mg base/kg is effective and well tolerated [2].

However, a relevant issue for health facilities in small communities of the Brazilian Amazon Basin is the unavailability of scales to weigh patients, and anti-malarial drugs are dispensed in small plastic bags at a dose regimen based on age. Therefore, it is relevant to evaluate whether children and adolescents are receiving suboptimal doses of chloroquine. Thus, the present study aimed to determine the dose administered in mg/kg and based on the BSA in order to estimate the extent of sub dosing of chloroquine in children and adolescents with vivax malaria using an age-based dose regimen. In addition, the influence of age on the plasma concentrations of chloroquine and desethylchloroquine was investigated.

## Methods

### Ethical statement

The study was revised and approved by the Ethical Committee of The Health Science Institute of the UFPA, Number 2.819.240.

### Study site

The present study was one component of a major research project designated to evaluate the nutritional status of children with vivax malaria on Marajo Island. It was carried out from January 2016 to May 2017 in the municipality of Anajas-Para, which is considered an area of high endemicity, with approximately 3000 annual cases of *P. vivax* in children and adolescents over the last 5 years.

### Participants

The inclusion criteria were male patients aged 2–14 years with slide-confirmed mono-infection with *P. vivax*. The exclusion criteria were patients with signs or symptoms of severe malaria (parasitaemia over 5%, jaundice, renal impairment, severe anaemia, altered level of consciousness), obesity, weight under 10 kg, known hypersensitivity or allergy to chloroquine, anti-malarial treatment within the 3 months prior to inclusion in the study, and those with relatives that did not give written informed consent.

### Treatment and follow-up

Patients were treated with chloroquine diphosphate tablets (Farmanguinhos Laboratory–Fundação Oswaldo Cruz/Rio de Janeiro, Brazil) in conjunction with primaquine diphosphate (Farmanguinhos Laboratory, Fundação Oswaldo Cruz/Rio de Janeiro, Brazil). The drugs were dispensed following the routine procedure of the health facility, which is in small plastic bags at a dose regimen based on patient age, following the recommendations of the Brazilian Health Authorities (Table 1) [17]. Drug administration was supervised by the researcher during all treatments, and the occurrence of vomiting was monitoring for 2 h following intake. A scale was provided by the research team to weigh the patients upon admission to the study. All patients were requested to return for a follow-up visit on days 1, 3, 5, 7, 14, 28, and 42 or at any time that signs and symptoms suggestive of malaria occurred [18].

**Table 1:** Chloroquine doses for treatment of uncomplicated vivax malaria recommended by the Brazilian Malaria Program Control [11]

Age	Body weight (kg)	Day 1^a^	Day 2^a^	Day 3^a^
1–3	10–14	150	75	75
4–8	15–24	150	150	150
9–11	25–34	300	300	300
12–14	35–49	450	300	300

^a^Tablets of chloroquine diphosphate with 150 mg of the base

### Parasite count

Parasite count was performed on Giemsa-stained thick blood films on each day of the follow-up visit. An experienced microscopy expert examined the blood films using 100× (oil immersion) objectives [18].

### Anthropometric measures and evaluation of chloroquine underdosing

The height and weight of each patient were collected at enrolment in the study. Weight was measured using a digital weight balance, and height was assessed by a stadiometer with a lateral scale in centimetres. The body surface area (BSA) was determined in children weighing over 10 kg by the Boyd formula, as follows [19]:

$$\text{BSA}\left( {\text{m}^{2} } \right) = 0.0003207 \times \text{height}\;\left( {\text{cm}} \right)\;0.3 \times \text{weight}\;\left( \text{g} \right) \times 0.7285 - \left[ {0.0188 \times \log \;\left( {\text{weight}} \right)} \right].$$

The total dose of chloroquine administered based on BSA was calculated using the following equation [8, 14, 20]:

$$\text{Total}\;\text{dose}\;\left( {{{\text{mg}} \mathord{\left/ {\vphantom {{\text{mg}} {\text{m}^{2} }}} \right. \kern-0pt} {\text{m}^{2} }}} \right) = \text{BSA} \times \text{chloroquine}\;\text{dose}\;{\text{administered}}$$

The required dose of chloroquine was determined using the following equation [8, 14, 20]:

$${\text{Required}}\;{\text{dose}}\;\left( {{{\text{mg}} \mathord{\left/ {\vphantom {{\text{mg}} {\text{m}^{2} }}} \right. \kern-0pt} {\text{m}^{2} }}} \right) = \left( {{{\text{BSA}} \mathord{\left/ {\vphantom {{\text{BSA}} {1.73}}} \right. \kern-0pt} {1.73}}} \right) \times \text{adult}\;\text{total}\;\text{dose}\;\text{of}\;\text{chloroquine}\;\left( {1500\;\text{mg}} \right)$$

A subdose of chloroquine was accepted when the amount of drug by body weight was below 25 mg/kg as well as when the total dose required, or optimal dose, based on BSA was above the administered dose.

### Blood sample collection and measurement of chloroquine and desethylchloroquine

Venous blood samples (4 ml) were taken for measurement of chloroquine and desethylchloroquine on day 7, which is approximately 168 h after the last dose of chloroquine. Following collection, the samples were centrifuged at 3000×*g* for 10 min at 4 °C for plasma separation. The samples were immediately stored at − 80 °C until analysis.

The concentrations of chloroquine and desethylchloroquine were measured using a reversed-phase HPLC system with fluorescence detection (Flexar, Perkin Elmer ™, Shelton, MA US) after liquid–liquid extraction as described by Alvan et al. with minor modifications [21, 22]. Briefly, 80 µl of internal standard (quinine 2.5 µg/ml), 1 ml of 1 M sodium hydroxide, and 6 ml of diethyl ether were added to 500 µl of plasma sample and mixed by shaking for 15 min. After vortex mixing for 30 s, the sample was centrifuged at 1000×*g* for 10 min, and the organic layer was separated and evaporated under a stream of nitrogen. The residue was resuspended in the mobile phase for HPLC analysis. The separation was carried out on a reversed-phase RP-18 column (X terra 4.6 × 150 mm, i.d. 5-μm) at 25 °C. The mobile phase consisted of dichloromethane-methanol-(1 M) perchloric acid (100:9:1.2; v:v:v) at a flow rate of 1.2 ml/min. The eluate was monitored by a fluorescence detector (*E*_*xcitation*_= 340 nm; *E*_*mission*_= 380 nm). The limit of detection for both analytes was 10 ng/ml, and the limit of quantification was 25 ng/ml for chloroquine and 30 ng/mL for desethylchloroquine. The assay was linear from 25 to 2000 ng/ml for chloroquine and from 30 to 2000 ng/ml for desethylchloroquine. Plasma samples spiked with chloroquine or desethylchloroquine at concentrations of 50, 200 and 500 ng/ml were used to estimate the recovery and intra- and inter-day coefficients of variation. The mean coefficients of variation for chloroquine were 8.3% and 10.5% and they were 10.3% and 14.7% for desethylchloroquine. The mean recovery was 95%. There was no significant interference by primaquine, carboxy-primaquine, mefloquine, carboxy-mefloquine, or acetaminophen in the detection of chloroquine and its main metabolite.

### Data analysis

The data are described as the median and range or as frequencies of occurrence. The normal distribution was assessed by the Kolmogorov–Smirnov test. The comparison of variables among age groups was performed using the Kruskal–Wallis H test. The Mann–Whitney U test was used to compare the total dose administered of chloroquine and the total dose required in each age group. All p-values were two-tailed, and p < 0.05 is considered statistically significant. Statistical analyses were performed using the STATISTICA software package (version 7.0; StatSoft Inc. 2004, Tulsa, USA).

## Results

A total of 81 male patients with *P. vivax* malaria were recruited and completed the study. The median age was 9 years old (2–14 years). All patients presented with negative blood smears at the 42-day follow-up visit suggesting that chloroquine is still effective against the asexual stage of *P. vivax* in this endemic setting. On day 3, there were no parasite in the peripheral blood of patients that received a chloroquine total dose of 25 mg/kg or above. However, 7 patients that received a total dose below 25 mg/kg from age groups of 2–3 (n = 4) and 4–8 years (n = 3) showed positive blood smears, with a geometrical mean of 81.4 (52–320) and 15 (10–32) parasites/µl, respectively. There was no report of vomiting during the course of treatment. However, most patients reported nausea. The nutritional status of the study participants is described elsewhere [23]. The baseline characteristics of the studied patients are shown in Table 2.

**Table 2:** Baseline characteristics of patients included in the study

Characteristic	2–3 (n = 22)	4–8 (n = 18)	9–11 (n = 19)	12–14 (n = 22)
Weight^a^, kg	12.6 (11.5–13.8)	19.4 (16.4–21.8)	33(31–36)	46 (40–52)
Body surface area^a^, m^2^	0.57 (0.54–0.60)	0.79 (0.70–0.8)	1.13 (1.09–1.2)	1.4 (1.29–1.5)
Parasitemia at admission, geometrical mean and standard deviation	4420 (3.64)	3637 (2.68)	1639 (3.39)	3528 (5.92)
Number of patients with parasitemia on day 3,	4	3	nd	nd
Parasitemia on day 3, geometrical mean and range	81.44 (52–320)	15 (10–32)	nd	nd
Parasite clearance^a^, h	60 (24–96)	48 (24–72)	48 (24–60)	48 (24–60)
Previous episodes of malaria, %	60	80	90	90
History of fever in the least 48 h, %	92	85	90	80
Time for fever clearance^a^, h	24 (12–48)	24 (12–60)	24 (12–60)	24 (12–60)
Hemoglobin^a^, g/dl	11.8 (10.9–12.5)	12.1 (11–13.5)	12.6 (11.1–12.8)	13.4 (12.1–14.5)
Hematocrit^a^, %	34 (29–37)	36 (29–38)	36 (29–39)	38 (31–40)
Platelets^a^, mm^3^	212,000 (190,000–280,000)	235,000 (160,000–270,000)	242,000 (185,000–310,000)	260,000 (195,000–320,000)

*Nd* not determined

^a^Values are expressed as median and range

The total dose of chloroquine administered based on mg/kg was normally distributed (*p *= 0.122), ranging from 13.1 mg/kg to 38.1 mg/kg. The percentage of patients with a total dose below 25 mg/kg ranged from 29.4 to 63.6% (*X*^2^ = 14.3; *p *= 0.0024).

**Fig. 1:**
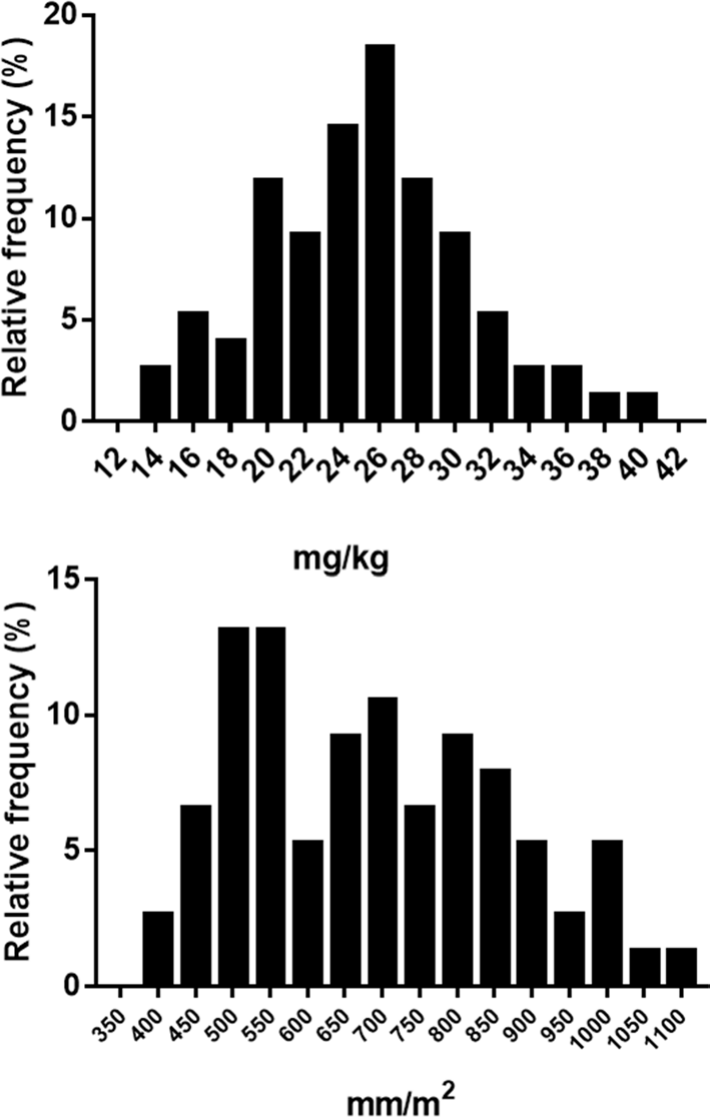
Frequency of distribution of chloroquine dose, expressed in mg/kg and in mm/m^2^

The total dose of chloroquine administered based on BSA ranged from 387 to 1079 mg/m^2^. The data were not normally distributed (*p *< 0.05) (Fig. 1). The values increased with age and were significantly different between the age groups (H = 57.3; *p *< 0.0001). The required total dose of chloroquine based on BSA ranged from 430 mg/m^2^ to 1552 mg/m^2^. The comparison between the required total dose and the administered dose based on BSA was similar in children aged 2–3 years (U = 193; *p *= 0.2549). However, the administered dose was lower than the optimal dose in children aged 4–8 years (U = 64; *p *= 0.007) and 9–11 years (U = 32; *p *< 0.001) and in adolescents aged 12–14 years (U = 18; *p *< 0.0001) (Table 3).

**Table 3:** Dose of chloroquine administered in each age group and the dose administered by body weight and body surface area

Age group (years)	n^a^	Dose administered (mg)	Dose administered^b^ (mg/kg)	Dose below 25 mg/kg, (%)	Dose required^b^ (mg/m^2^)	Dose administered^b^ (mg/m^2^)
2–3	22	300	23.7 (16.9–29.7)	63.6	502 (430–631)	517 (412–602)
4–8	18	450	23.5 (13.4–38.1)	56.25	688 (508–1007)	566 (387–766)
9–11	19	900	26.7 (16.1–34.6)	29.4	986 (751–1296)	736 (650–998)
12–14	22	1150	22.8 (13.1–32.2)	61.9	1108 (843–1552)	917 (669–1079)

^a^Number of patients

^b^Values are expressed as median and range

**Fig. 2:**
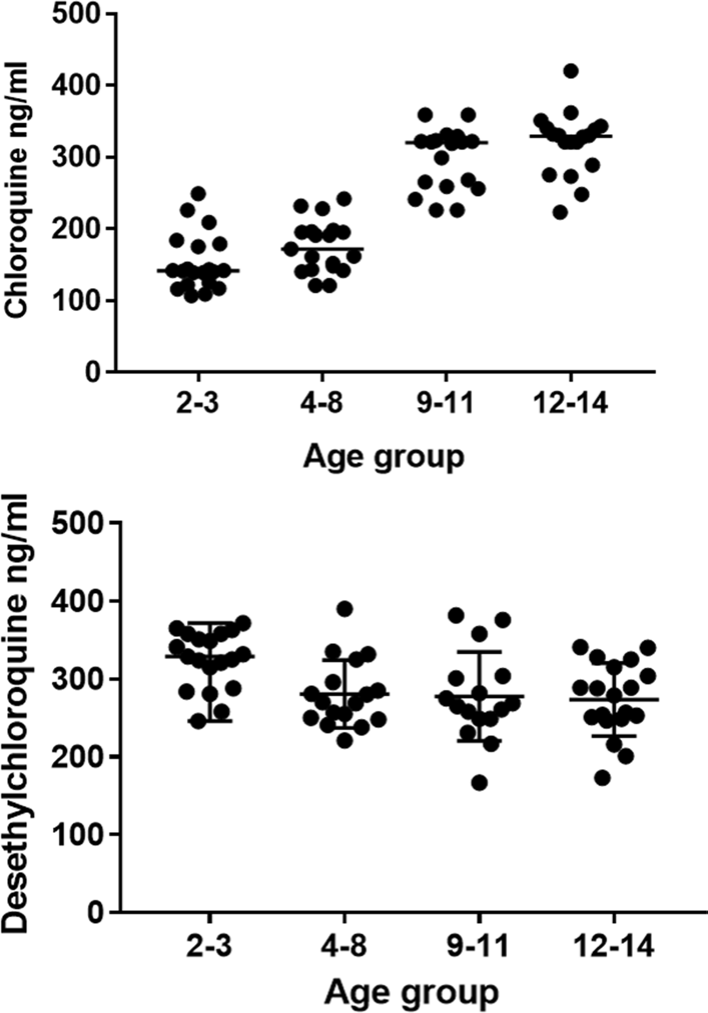
Plasma concentrations of chloroquine and desethylchloroquine

The median time for collection of blood samples for drug analysis was 168 h (166–169 h). Plasma chloroquine concentrations ranged from 107 to 420 ng/ml, and the median values increased significantly with age (H = 22.8; *p *< 0.001). For desethylchloroquine, the plasma concentrations ranged from 167 to 390 ng/ml, with a similar median value among age groups (H = 3.74; *p *= 0.2905) (Fig. 2).

## Discussion

Accurate dosing contributes to the therapeutic efficacy of anti-malarial drugs, and children constitute a group at risk for suboptimal dosing, since organ maturation, body composition, and the ontogeny of drug elimination pathway affect the disposition of these drugs [24, 25]. Moreover, vomiting, diarrhoea, malnutrition and other comorbidities, pharmaceutical formulation and ethnicity can modify drug exposure, increasing the risk of suboptimal dosing of anti-malarial drugs [7, 26, 27].

In the current study, subdosing of chloroquine was assessed by both the dose administered, calculated in mg/kg, and by the difference between the required or optimal dose and the administered dose based on BSA. Only male patients were included in the study in order to avoid potential influence of differences between genders related to physical growth and sexual maturation especially in older children and adolescents [13, 24, 25].

In Brazil, the official guidelines for the treatment of malaria for children and adolescents below 15 years of age recommend a dose regimen of chloroquine based on age, or preferably on body weight [17]. Due to operational reasons, anti-malarial drugs are dispensed as an age-based dose regimen in most endemic municipalities. Despite the advantages of fixed dose for a specific age group, as ease of administration, lower risk of medical errors, and cost-effectiveness, this dosing regimen of anti-malarial drugs does not take into account the variation of body weight as well as in the metabolism and clearance in each age range, and thus it is often inaccurate and may lead to a lack of efficacy or toxicity [12, 13, 25].

In fact, when the aged-based total dose of chloroquine was transformed to milligrams of drug per kilogram of body weight (mg/kg), there was a normal distribution of the data demonstrating a high variation in chloroquine doses. Moreover, there was an elevated proportion of patients receiving a suboptimal dose of the drug (< 25 mg/kg), which ranges from 27.5 to 60.5% in the age groups investigated, with the highest proportion in children aged 2–3 years old, which corroborates reports of the high risk of under dosing in young children [7–10].

The highest total dose of chloroquine administered was 38.1 mg/kg. A serious toxic effect in patients receiving such a high chloroquine dose is improbable, because previous studies have shown the safe use of chloroquine doses of 50 mg/kg in African children with *P. falciparum* malaria [28].

The suboptimal dose of chloroquine was also assessed by the difference between the required and the administered dose of the drug based on BSA, which is considered the better index to dose normalization in children, since BSA consider the variation in age, body size and body composition [9, 13, 14, 16, 20]. The BSA-based total dose of chloroquine administered to the patients did not show a normal distribution, which is consistent with the non-linear increase in BSA among the age groups [12, 20]. As expected, BSA-based total chloroquine doses increased with patient age. The differences between the optimal and the administered doses of chloroquine revealed that the age groups of 4–8, 9–11 and 12–14 years old received suboptimal doses of the drug. This finding corroborates the data from weight-based doses and are consistent with those of previous studies that chloroquine is under-dosed in children and have suggested an increase in the chloroquine dose or an adjustment of the dose based on BSA [8–10].

However, in children aged 2–3 years old, the BSA-based dose regimen demonstrated adequate dosing of chloroquine, which disagrees with the obtained results of doses estimated in mg/kg in this age group with the highest proportion of under dosing. Such a discrepancy is due to the shortcomings of BSA calculation in young children and neonates, in which dose adjustment based on body weight is preferable. As a rule, the BSA is not calculated in children younger than 2 years or weighing less than 10 kg [15, 16, 19, 20]. Thus, the median weight of 12.6 kg in the 2–3 year-old patients probably classifies this group as borderline for the accurate estimation of BSA.

The influence of age on the plasma concentrations of chloroquine and desethylchloroquine was assessed in blood samples collected on day 7, which provides a reliable assessment of parasite exposure to the drug [29]. The plasma concentrations of chloroquine and desethylchloroquine found in the present study patients are in consistent with those reported in children and adolescents under a similar dose regimen in other endemic areas [8, 9].

The concentrations of chloroquine in the plasma varied significantly with age, with the highest values being found in adolescents aged 12–14 years old. This result confirms that age has a significant influence on the exposure of *P. vivax* to the drug. In fact, the WHO states that the recommended total dose of chloroquine of 25 mg/kg leads to a lower drug concentration in young children and infants than that in older children [2]. On the other hand, the plasma concentrations of desethylchloroquine decreased with age, suggesting a decrease in chloroquine clearance in older children and adolescents, which corroborates the high drug clearance found in children with uncomplicated malaria compared with healthy adults [30, 31].

As expected, suboptimal doses of chloroquine (< 25 mg/kg) delayed the clearance of parasites in 30% of studied patients from age groups of 2–3 and 4–8 years [7, 10]. Despite the widespread suboptimal dose of chloroquine and the age-related variation of chloroquine plasma concentrations, all patients presented an adequate therapeutic response at day 42. An adequate therapeutic efficacy of chloroquine has previously been reported at doses below 25 mg/kg [32]. Moreover, a potential influence of the concurrent administration of primaquine to improve the schizontocidal efficacy of chloroquine should contribute to the adequate therapeutic response at day 42 [10, 33].

These data support the importance of clinical trials and short-term in vitro assays to investigate the accurate total dose of chloroquine that prevents exposure to sub-therapeutic concentrations or toxicity in different ages and population groups. The considered optimal dose of 25 mg/kg was determined in the 1940s and has suffered minor modifications during its 79 years of use [9].

The limitations of the study were the small number of patients included in each group as well as the adverse reactions were not evaluated in the study patients.

## Conclusion

This is the first study in the Brazilian Amazon Basin to evaluate the extent of sub-optimal doses of chloroquine in children and adolescents with malaria by *P. vivax*. Based on the optimal dose referenced by the WHO (25 mg/kg), in addition to the difference between the optimal and those administered doses based on BSA, suboptimal doses of chloroquine were found in all of the age groups investigated. The data of the study highlight the necessity of clinical trials and short-term in vitro assays to investigate the accurate total dose of chloroquine that prevents exposure to sub-therapeutic concentrations or toxicity in different ages and population groups. Moreover, the data show the widespread use of sub-optimal doses of chloroquine in children and adolescent from the referred endemic area which highlight the requirement of efforts to abolish the dose regimen based on patient age.

## Data Availability

The datasets used and/or analyzed during the current study are available from the corresponding authors on reasonable request.

## Abbreviations

WHO: World Health Organization
BSA: body surface area
HPLC: high-performance liquid chromatography
UFPA: Para Federal University
